# Adipose and Muscle Tissue Gene Expression of Two Genes (*NCAPG* and *LCORL*) Located in a Chromosomal Region Associated with Cattle Feed Intake and Gain

**DOI:** 10.1371/journal.pone.0080882

**Published:** 2013-11-20

**Authors:** Amanda K. Lindholm-Perry, Larry A. Kuehn, William T. Oliver, Andrea K. Sexten, Jeremy R. Miles, Lea A. Rempel, Robert A. Cushman, Harvey C. Freetly

**Affiliations:** United States Department of Agriculture, Agricultural Research Service, United States Meat Animal Research Center, Clay Center, Nebraska, United States of America; Institute of Farm Animal Genetics, Germany

## Abstract

A region on bovine chromosome 6 has been implicated in cattle birth weight, growth, and length. Non-SMC conodensin I complex subunit G (*NCAPG*) and ligand dependent nuclear receptor corepressor-like protein (*LCORL*) are positional candidate genes within this region. Previously identified genetic markers in both genes were associated with average daily gain (ADG) and average daily feed intake (ADFI) in a crossbred population of beef steers. These markers were also associated with hot carcass weight, ribeye area and adjusted fat thickness suggesting that they may have a role in lean muscle growth and/or fat deposition. The purpose of this study was to determine whether the transcript abundance of either of these genes in cattle adipose and muscle tissue was associated with variation in feed intake and average daily gain phenotypes. Transcript abundance for *NCAPG* and *LCORL* in adipose and muscle tissue was measured in heifers (adipose only), cows and steers using real-time polymerase chain reaction. In the adipose tissue from cows and heifers, a negative correlation between *LCORL* transcript abundance and ADFI were detected (*P* = 0.05). In the muscle tissue from cows, transcript abundance of *NCAPG* was associated with ADG (r = 0.26; *P* = 0.009). A positive correlation between *LCORL* transcript abundance from muscle tissue of steers and ADFI was detected (*P* = 0.04). LCORL protein levels in the muscle of steers were investigated and were associated with ADFI (*P* = 0.01). These data support our earlier genetic associations with ADFI and ADG within this region and represent the potential for biological activity of these genes in the muscle and adipose tissues of beef cattle; however, they also suggest that sex, age and/or nutrition-specific interactions may affect the expression of *NCAPG* and *LCORL* in these tissues.

## Introduction

Genetic markers located within the genes *NCAPG* and *LCORL* have been implicated in several QTL and genome-wide association studies for human height, swine body length, equine height, chicken carcass weight, and also for cattle growth phenotypes [1]–[12]. Moreover, numerous cattle QTL for birth weight, growth and length have been identified within or near the *NCAPG-LCORL* loci [13]–[17]. In addition to phenotypes related to size and growth, this region appears to contribute to ribeye area and subcutaneous fat [4], [5], [17] suggesting a potential mechanism through muscle growth and/or lipid deposition.

In cattle, the *NCAPG-LCORL* gene locus is located on BTA6 between 38.76 to 38.99 Mb on the UMD 3.1 genome assembly. *NCAPG* is a subunit of the condensin 1 protein involved in chromatin condensation during replication. *NCAPG* may also have a role in modulating fetal growth in cattle [6]. A mutation at *NCAPG* c.1326T>G alters the coding amino acid at position 442 from an isoleucine to a methionine (p.I422M) and has been implicated in cattle growth in three populations of cattle [5]–[6].

Two studies have evaluated whether there is a biological or functional role for NCAPG in cattle growth [6], [18]. Eberlein et al. [6] detected *NCAPG* expression in skeletal muscle, lung, brain, bone, fetal and maternal placental tissue and examined the effect of the p.I422M loci on fetal growth. *NCAPG* transcript abundance was measured with quantitative RT-PCR in the fetal portion of six placentomes with differing genotypes at the p.I422M locus. The results were suggestive of a genotypic effect on transcript level (*P* = 0.09). A metabolomics study by Weikard et al. [18] concluded that the p.I422M polymorphism in *NCAPG* was associated with circulating levels of plasma arginine, symmetric dimethylarginine (SDMA), and linoleylcarnitine (C18∶2) levels. Arginine affects growth through activation of the mTOR pathway [19] and is also a precursor for nitric oxide. These data suggest a physiological role for *NCAPG* on growth through arginine; however, the mechanisms of its role in this pathway have not yet been identified.

We have also previously shown that the p.I422M polymorphism was associated with ADG and ADFI in our population of crossbred cattle. However, SNP further downstream and within the *LCORL* gene locus showed more highly significant associations and two of these were validated in an unrelated crossbred population of cattle supporting a potential role for *LCORL* in beef steer gain [4]. Current literature with biological information regarding the *LCORL* gene is limited. *LCORL* is thought to be a transcription factor that may function during spermatogenesis in the testes. There is strong evidence that polymorphisms in the *LCORL* gene are associated with human skeletal frame size and height [1]–[3] and with equine height at withers [7]–[10], although there is no information regarding the potential functional mechanisms of *LCORL* in height and growth. A recent study examining *LCORL* gene expression in horse hair follicles detected a correlation between transcript abundance and the genotypes of an SNP in *LCORL* associated with height at withers [9].

Since differential expression of both *NCAPG* and *LCORL* has been correlated to polymorphisms [6], [9], [18] and genetic markers in both genes have been associated with economically important cattle production traits [4]–[6], we chose to further evaluate these genes for expression in different groups of cattle of various sex, ages and diets. The purpose of this study was to determine whether transcript abundance of either *NCAPG* or *LCORL* in adipose and muscle tissue was associated with growth and/or feed intake traits in various beef cattle models.

## Methods

### Animals

The U.S. Meat Animal Research Center (USMARC) Animal Care and Use Committee reviewed and approved all animal procedures. The procedures for handling the cattle complied with the Guide for the Care and Use of Agricultural Animals in Agricultural Research and Teaching [20]. Biopsies were performed with local lidocaine blocks and all efforts were made to minimize animal suffering.

### Populations of cattle

Heifers and cows. The breeding plan for the heifer and cow populations used in this study has been described previously [21], [22]. Purebred Angus, Hereford, Simmental, Limousin, Charolais, Gelbvieh and Red Angus sires were mated with MARC III (¼ Angus, ¼ Hereford, ¼ Pinzgauer, ¼ Red Poll), Angus or Hereford cows using artificial insemination to yield F_1_ offspring that were born during 1999, 2000, and 2001. Female F_1_ animals born in 1999 and 2000 and F_1_ males born in 2001 were mated in pastures with multiple sires to produce 2-, 3- and 4-breed progeny F_1_^2^ animals. The F_1_^2^ calves were born to dams of 3 years of age and older in the spring from 2003 to 2007. Calves were weaned during September at roughly 165 d [22]. Heifers used in this study were F_1_^2^ animals born in 2006 and 2007; cows were 2005- and 2006-born animals.

Steers were born in either the spring or fall of 2011 and were from the USMARC continuous Germplasm Evaluation Program [23]. These animals were part of a breed expansion plan to produce purebred cattle of 16 breeds. The breeds include the seven mentioned above and also Brangus, Beefmaster, Braunvieh, Shorthorn, Brahman, Maine Anjou, Santa Gertrudis, Salers, and Chi-Angus. Sires from each of the 16 breeds were mated to female offspring from cattle from the population described above. Animals were removed from the study when diagnosed with conditions that could impact feed intake and gain phenotypes, like pneumonia, foot-rot, and bloat.

### Feed efficiency phenotypes

Heifers were trained on the test ration beginning at 276±15 d of age and were on the study for 84 days. Individual feed intake measurements were obtained from Calan Broadbent Feeding Systems (American-Calan-Broadbent, Northwood, NH). At 0800 h, animals were given *ad libitum* access to feed. The dry matter mixed ration included 64.8% corn silage, 30% ground alfalfa hay, 5% soybean meal, and 0.2% sodium chloride. Feed offered was recorded daily and feed refusals were collected weekly.

Feed efficiency phenotypes for heifers were collected as follows: dry matter intake (DMI) was equal to cumulative dry matter intake for the 84-d feeding period. Average daily feed intake is the total dry matter intake (DMI) divided by 84 (DMI/84). Individual animal quadratic regressions for time on study were fit for body weight on time (weighed every 2 weeks), and gain was calculated as the difference of BW predicted at 84 d and the intercept. Average daily gain was the calculation for total gain over the study period divided by days on feed.

Cows were not pregnant or lactating. Approximately 1 month after their last calf was weaned (cow age was 5.58 years ±47 d) cows were fed a diet to achieve 120 kcal of metabolizable energy per unit of body weight^0.75^ per day for 98 d. The diet consisted of 67.3% corn silage, 27% alfalfa hay, 5.5% corn, and 0.2% salt. Subsequently, cows were allowed *ad libitum* access to the same feed. Biopsies were performed 6 weeks after the animals had *ad libitum* access to feed. Feed efficiency phenotypes for cows were collected as described for heifers except the length of the study was 98 d after *ad libitum* feeding was restarted.

Steers (n = 188) born in the spring of 2011 and 139 steers born in the fall of 2011 were evaluated for feed efficiency using an Insentec system (Marknesse, The Netherlands) over 63-d and 92-d periods, respectively. The age of steers at the beginning of the trials was 344±48 d. Spring-born steers were given *ad libitum* access to a ration consisting of 57.75% high-moisture corn, 30% wet distillers grain with soluble (WDGS), 8% alfalfa hay, and 4.25% Steakmaker® with monensin (Land O' Lakes, Inc, Gray Summit, MO). Fall-born steers were given *ad libitum* access to a ration consisting of 57.35% dry-rolled corn, 30% WDGS, 8% alfalfa hay, 4.25% Steakmaker, and 0.4% urea. Gain over the test period was calculated by quadratic regression, and DMI was equal to the total cumulative dry matter intake over the same period. ADFI and ADG were calculated as described above for heifers except with the appropriate length of trial (either 63 or 92 d) in the denominator. Steers were screened and excluded for medical or health issues that may have affected either feed intake or gain phenotypes. Average daily gain was plotted on ADFI for each season and the four most extreme animals (i.e. 4 animals with high ADG and ADFI, 4 with high ADG and low ADFI, 4 with low ADG and low ADFI, and 4 animals with low ADG and high ADFI phenotypes were selected) were selected, for a total of 32 animals, for tissue sampling from each of the four Cartesian quadrants relative to the mean of the two traits (range of phenotype ADFI: 6.8–17.3 kg/d; ADG: 1.0–2.4 kg/d).

### Tissue collection and RNA isolation

Heifer adipose tissue was collected from animals born in 2006 and 2007 at 360±40 d of age. A lidocaine block (20 mL) was given and subcutaneous adipose tissue was removed from heifers through an incision approximately 10 cm below the tailhead. Samples were immediately stored at −80°C until RNA extraction was performed. The RNeasy Lipid Kit (Qiagen, Valencia,CA) was used according to the manufacturer's instructions to extract total RNA from 50–100 mg of adipose tissue from 94 heifers.

Cow adipose tissue biopsies were collected on 2005- and 2006-born animals at ∼6-years of age and muscle tissue biopsies were also collected from 2005-born animals. After creating a lidocaine block (20 mL), a small incision was placed approximately 10 cm down from the tailhead. Subcutaneous adipose was biopsied. Muscle tissue was biopsied from the same incision site using a 6 G×12.065 cm U.C.H Skeletal Muscle Biopsy Needle (Dyna Medical Corp, Ontario, Canada). Muscle samples were immediately stored at −80°C until RNA extractions were performed. Total RNA was extracted from 50–100 mg of tissue with TriZol (Invitrogen, Carlsbad, CA) according to the manufacturer's instructions with the exception that the centrifugation step subsequent to adding chloroform to the homogenized tissue was increased from 15 to 20 min.

Steers. Adipose tissue from the rump and muscle tissue from the longissimus dorsi were collected from 2011-born steers with extreme ADG and ADFI phenotypes at slaughter (440±31 d of age). Tissues were collected between 10 and 45 min post mortem. Samples were diced and immediately frozen in liquid nitrogen. RNA was extracted using TriZol with the same exception as described above for the cows.

Total RNA was quantified with a NanoDrop 8000 spectrophotometer (Wilmington, DE). RNA (2 µg) was reverse transcribed using ReadyMade random hexamer primers (Integrated DNA Technologies, Coralville, IA) and M-MLV reverse transcriptase (Promega, Madison, WI) according to the manufacturer's protocol.

### Gene expression

Primers used to amplify *NCAPG* and *LCORL* from cDNA were located in exons 3 and 4 in both genes. *NCAPG* forward primer sequence was 5′- AGG ATA CAG GCA GTT CTT GCT C-3′ and the reverse primer sequence was 5′- ATA ACA CTG CCC GCC TAA CTT -3′. The forward primer sequence for *LCORL* was 5′- GTG AAC CAG AAG AGC TGA CTG A -3′ and the reverse primer sequence was 5′- GTT CCT CTG TTG GTG TTG ACT G -3′. The reference or internal control gene used was the *protein kinase C inhibitor protein 1*, also known as *tyrosine 3-monooxygenase/tryptophan 5-monooxygenase activation protein, gamma polypeptide* (*YWHAG*). The YWHAG forward primer sequence was 5′- GCG AGA CCC AGT ATG AGA GC -3′ and the reverse primer sequence was 5′- AAG GGC CAG GCC TAA TCT AA -3′. We were unable to identify a stable housekeeping gene for adipose tissue from the steers. In lieu of a housekeeping gene, a standard curve was generated with a standard serial dilution of pooled cDNA consisting of 100 ng, 50 ng, 25 ng 12.5 ng, 6.25 ng and 3.125 ng equivalents of cDNA to evaluate the expression of *NCAPG* and *LCORL* in these tissue samples.

Real-time PCR was performed in triplicate with 1X QuantiTect SYBR Green PCR Master Mix (Qiagen), 1 µL of 50 ng/µL cDNA template from heifer and cow samples, and 0.48 µM each primer. The PCR reactions were performed at 95°C for 5 min followed by 41 cycles at 95°C for 10 s, 58°C for 20 s, and 80°C for 1 s with a final melting curve from 65 to 95°C on a MJ PTC-200 with a Chromo-4 detector (MJ Research, Watertown, MA). A pooled control sample served as a calibrator sample for all primer sets on each plate.

Real-time PCR on steer tissue samples was performed in triplicate with Roche LightCycler® 480 SYBR Green I Master mix (Roche Molecular Biochemical, Indianapolis, IN), 1 µL of a 1∶20 dilution of 50 ng/µL cDNA template from muscle or 1 µL of a 5 ng/uL cDNA template from adipose tissue, and 0.48 µM each primer. The PCR reaction was performed at 95°C for 5 min followed by 45 cycles of 95°C for 10 s, 60°C for 10 s, 72°C for 10 s, and a final melting curve from 65 to 95°C. The threshold cycle (Cp) for *NCAPG, LCORL* and *YWHAG* from each sample was determined and used to calculate the ΔΔCt using the reference pooled cDNA samples (Note: *YWHAG* was not used for steer adipose samples, instead a standard curve method was used [24]). The fold change expression differences between samples were calculated with the 2^−ΔΔCt^ method [25]. Calculation of relative quantity of expression for the steer adipose tissues were calculated using a relative standard curve method by plotting treatment Cps against the logarithmic values of standard amounts of pooled cDNA [24].

### Western immunoblots

Muscle samples were analyzed for LCORL abundance by Western blot analysis. Muscle was powdered at liquid nitrogen temperature and a crude muscle soluble protein extract was prepared in buffer that contained 50 mM Tris HCl (pH 7.5); 3 mM EDTA; 100 mM NaCl; protease inhibitors (Complete Mini, 1836153, Roche Applied Science, Indianapolis, IN); and 1% IGEPAL A-630 (USB Corp., Cleveland, OH). The protein concentrations of the extracts were determined using the bicinchoninic acid reagent [26]. Soluble protein (40 µg) was resolved on 10% SDS-PAGE gels and electroblotted onto polyvinylidene fluoride membranes (Pall Co., Pensacola, FL). The membranes were probed for total LCORL with rabbit anti-human LCORL antibody (1∶2,000; NBP1-916, Novus Biologicals, Littleton, CO).

### Gene and protein expression data analyses

All analyses were conducted using MTDFREML [27] or SAS 9.3 (Cary, NC). Correlations were derived for all expression and phenotypic data within the cow, heifer, and steer data sets as a preliminary analysis.

Cow and heifer ADG and ADFI were analyzed in a mixed model with fixed effects of year and fixed covariates for breed percentage, heterosis, and individual expression level (transcript abundance for adipose or muscle tissue) as covariates and random effect of animal with pedigree relationships among the cows and heifers.

Steers. Due to limited numbers of animals for each breed, breed effects and animal relationships were not fitted in the analysis for the steer data set. Because extreme animals for ADG and ADFI were selected from each of four Cartesian quadrants, expression data (transcripts and protein) was modeled with fixed effects of season and quadrant. Linear contrasts were then applied to quadrant to separate whether expression varied by low vs. high ADG, low vs. high ADFI, or their interaction. This analysis with expression as the independent variable was necessary to ensure the covariates for expression from the first model were not biased due to choosing steers from quadrant extremes.

## Results

A summary of the phenotypic and the *NCAPG* and *LCORL* gene expression data is provided in Table 1 for each population of animals. Values are given for the range, mean and standard deviation.

**Table 1:** Description of phenotypic data from heifers, cows and steers.

Animals	Phenotype^1^	Minimum	Maximum	Mean	SD
Heifer Population^2^	ADG	0.5	1.4	1	0.2
	ADFI	5.5	10.4	7.8	1
					
	Adipose *NCAPG*	0.04	14.4	1.3	2
	Adipose *LCORL*	0.05	10.8	1.1	1.3
Cow Population^3^	ADG	1.0	2.3	1.7	0.2
	ADFI	12.9	22.2	18.1	2.1
					
	Adipose *NCAPG*	0.1	5.9	1	0.8
	Adipose *LCORL*	0.4	2.2	1.1	0.4
Cow Population^4^	ADG	0.9	2.4	1.7	0.3
	ADFI	11.9	22.3	18	2.1
					
	Muscle *NCAPG*	0.4	2.4	1	0.4
	Muscle *LCORL*	0.6	2.6	1.1	0.4
Steer Population	ADG	1	2.4	1.8	0.4
	ADFI	6.8	17.3	10.6	2.5
					
	Adipose *NCAPG* 5	0.4	1.5	0.9	0.5
	Adipose *LCORL* 6	0.1	1.8	1	0.5
	Muscle *NCAPG*	0.1	1.9	0.8	0.4
	Muscle *LCORL*	0.6	1.4	0.9	0.2

1 ADG =  Average daily gain (kg/d); ADFI =  Average daily feed intake (kg/d); Adipose *NCAPG* =  fold change expression values for *NCAPG* from adipose tissue; Adipose *LCORL* = fold change expression values for *LCORL* from adipose tissue; Muscle *NCAPG* =  fold change expression values for *NCAPG* from muscle tissue; Muscle *LCORL* =  fold change expression values for *LCORL* from muscle tissue.

2 *NCAPG* and *LCORL* gene expression evaluated in 2006- and 2007-born heifers (n = 94).

3 2005- and 2006-born cows tested for gene expression from adipose tissue (n = 81).

4 2005-born cows muscle tissue samples tested for gene expression (n = 86).

5 Expression values based on 30 animals, 2 animals had negligible expression of *NCAPG* in adipose.

6 Expression values based on 31 animals, 1 animal had negligible expression of *LCORL* in adipose.

### Transcript abundance in heifers

Adipose tissue collected from heifers at approximately 1 year of age was examined for *NCAPG* and *LCORL* (n = 94) transcript abundance using real-time PCR. The transcript abundance of each gene was analyzed separately for effects on ADFI or ADG. *LCORL* transcript abundance was associated with ADFI (*P*≤0.05; Table 2).

**Table 2:** Gene expression in heifer adipose tissue and association and estimated effects for ADFI and ADG.

	ADFI^1^		ADG^2^	
*Gene*	*Effect (SE)* 3	*P-value*	*Effect (SE)* 3	*P-value*
*NCAPG*	−1.04 (±0.52)	0.051	0.0079 (±0.0089)	0.38
*LCORL*	**−1.06 (±0.53)**	**0.045**	0.020 (±0.013)	0.12

1 ADFI =  Average daily feed intake (kg/d).

2 ADG =  Average daily gain (kg/d).

3 Estimated effect in kg/d and standard error of unit of transcript abundance in fold change of *NCAPG* or *LCORL* on either ADFI or ADG.

### Transcript abundance in cows

Cows in this study were fed a restricted diet and then were placed on *ad libitum* access to feed for 12 weeks. Adipose (n = 81) and muscle (n = 86) tissue samples were examined for *NCAPG* and *LCORL* mRNA levels. Transcript abundance was analyzed for correlation with ADFI and ADG phenotypes (Table 3). Associations between the expression of *LCORL* and ADFI in adipose tissue (*P* = 0.02) and between ADG and *NCAPG* gene expression (*P* = 0.009) in the muscle tissue of these animals were identified.

**Table 3:** Gene expression in the muscle and adipose tissue of cows and their association and estimated effects for ADFI and ADG.

	ADFI^1^		ADG^2^	
	Effect (SE)^3^	*P-*value	Effect (SE)^3^	*P*-value
Muscle				
*NCAPG*	−0.52 (±0.017)	0.8	**0.26 (±0.096)**	**0.009**
*LCORL*	0.73 (±0.60)	0.2	0.060 (±0.096)	0.5
Adipose				
*NCAPG*	0.050 (±0.28)	0.9	0.011 (±0.045)	0.8
*LCORL*	**−1.54 (±0.66)**	**0.02**	−0.072 (±0.11)	0.5

1 ADFI =  Average daily feed intake (kg/d).

2 ADG =  Average daily gain (kg/d).

3 Estimated effect in kg/d and standard error of unit of transcript abundance in fold change of *NCAPG* or *LCORL* on either ADFI or ADG.

### Transcript abundance in steers

Longissimus dorsi muscle and subcutaneous adipose tissue from 32 steers with extreme feed efficiency phenotypes were collected. Tissues were collected over two seasons (spring and fall of 2012) from animals within four extreme phenotypic quadrants. Transcript abundance of *NCAPG* and *LCORL* was assessed. Expression was tested in a preliminary analysis for correlation with ADG and ADFI. No significant correlations were detected in the adipose tissue. However, the expression of *LCORL* in muscle was significantly correlated with ADFI (r = 0.38; *P* = 0.03). The transcript data was analyzed using gene expression as the dependent variable. The transcript abundance of *LCORL* in the muscle tissue was affected by intake (*P* = 0.04; Table 4).

**Table 4:** LSMeans and standard errors for gene or protein expression and phenotypes in the muscle and adipose tissue of steers.

	Extreme Phenotype^1^				*P*-value		
	H_I_H_G_	L_I_H_G_	L_I_L_G_	H_I_L_G_	Gain	Intake	Interaction
Muscle							
*NCAPG* 2	1.46 (±0.4)	1.18 (±0.4)	1.99 (±0.4)	1.66 (±0.4)	0.21	0.95	0.45
*LCORL* 2	0.99 (±0.08)	1.22 (±0.08)	1.27 (±0.08)	1.16 (±0.08)	0.19	0.04	0.45
LCORL protein^3^	226 (±44)	380 (±47)	301 (±47)	211 (±44)	0.30	0.01	0.49
Adipose							
*NCAPG* 2	0.96 (±0.12)	0.88 (±0.12)	1.01 (±0.12)	0.89 (±0.12)	0.77	0.87	0.41
*LCORL* 2	1.08 (±0.15)	0.84 (±0.15)	1.28 (±0.15)	0.96 (±0.15)	0.32	0.80	0.08

1 Extreme phenotypes: H_I_H_G_ =  high intake, high gain; L_I_H_G_ =  low intake, low gain; L_I_L_G_ =  low intake, low gain; H_I_L_G_ =  high intake, low gain. Data is shown as LSMeans with standard errors in parentheses.

2 NCAPG and LCORL gene expression units shown as fold change values.

3 LCORL protein units expressed as adjusted intensity units.

### Protein expression of LCORL in steers

**Figure 1:**
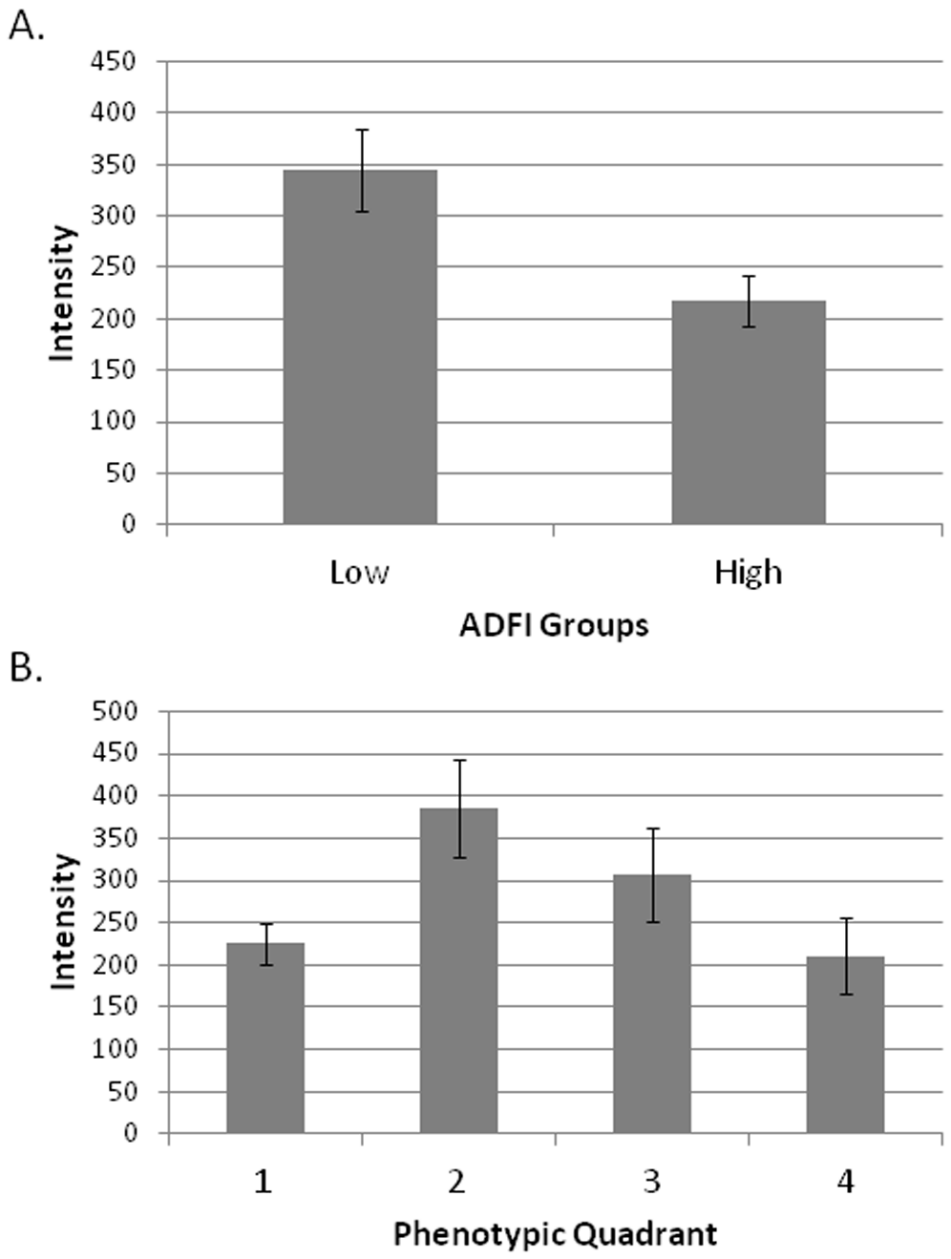
LCORL protein levels in steer muscle tissue measured by Western blot. A. LCORL protein levels by high and low ADFI groups. B. LCORL protein levels by ADFI and ADG phenotypic quadrants. Quadrant 1: high ADFI/high ADG, quadrant 2: low ADFI/high ADG, quadrant 3: low ADFI/low ADG, and quadrant 4: high ADFI/low ADG.

Protein expression of LCORL was examined in the muscle tissue from steers (n = 32) by Western blot. Two animals were removed from the analysis as outliers because their adjusted intensity values were greater than 2 SD from the mean. LCORL protein levels were significantly correlated with ADFI (*P* = 0.009) and for phenotypic quadrant (*P*<0.05, Figure 1). When protein was used as the dependent variable, the levels of LCORL protein in the muscle tissue are affected by intake (*P* = 0.01; Table 4).

## Discussion

There is growing evidence that the genes *NCAPG* and *LCORL* may have biological or functional roles in the growth or height phenotypes of species including cattle and horses [6], [9], [18]. Eberlein et al. [6] showed a trend between *NCAPG* transcript abundance and the *NCAPG* pI442M genotype. Animals with one or two copies of the T allele (c.1326T) had greater expression in the placenta. A metabolomics study by Weikard et al. [18] found that arginine plasma metabolites were associated with the p.I442M *NCAPG* marker in cattle suggesting a role in growth via the mTOR and/or the nitric oxide synthesis pathways. Additionally, Metzger et al. [9], analyzed expression of *NCAPG*, *LCORL* and *DCAF16* from horse hair follicles for relationships with a known *LCORL* genotype associated with height at withers. The relative transcript abundance of *LCORL* was lower in animals with one or two copies of the C allele, which potentially disrupts a TFIID transcription binding site and was associated with taller animals. Furthermore, previous SNP association data from our lab suggested that these genes may be involved in ADFI and ADG phenotypes via lean muscle growth and/or fat deposition [4].

Previously published studies carried out on the *NCAPG-LCORL* loci include a variety of mammalian species that differed by age and sex. Human GWAS studies for skeletal height and skeletal growth phenotypes have been performed on a female population age 50–79 years [3], mixed gender populations with measurements taken over ages 0–20 years [2], and ages 0–84 years [1]. These studies all identified associations between height phenotypes and SNPs within the *LCORL* gene. While association studies in male populations of Japanese cattle ages 2–2.5 years [9] and 6–21 months [28] have indicated that a non-synonymous SNP in the *NCAPG* gene is involved with carcass weight and body frame size. Similarly, studies on crossbred steers [20] and on a steer and heifer population of crossbred cattle between 9 months-1 year of age [19] identified this region, but not a specific gene, as associated with growth, intake and feed efficiency. In horses, the *NCAPG*-*LCORL* loci was associated with height at withers in a stallion population that was ∼2.5 years old [10] and SNPs specifically within the *LCORL* gene were associated with the same trait identified in two populations of horses of mixed sex and ages 4–34.5 years [8] or 1–10 years [9]. The aim of the current study was to determine whether the expression of either gene in muscle and adipose tissues could be correlated with feed intake and gain in three groups of cattle differing in age, sex and stage of life.

Our expression data illustrates unique differential gene expression correlated to phenotypes in both tissues in all three groups of cattle tested (heifers, steers and cows). A significant correlation between *LCORL* transcript abundance in adipose and ADFI was identified in heifers, and also in mature cows with *ad libitum* access to feed after feed restriction. Additionally, the cow population showed a positive association between muscle *NCAPG* transcript abundance and gain, whereas, the steers displayed correlations between gene and protein expression levels of *LCORL* and ADFI in the muscle. The data collected from steers in the current study, supports our previous work [4] that showed SNP within the *LCORL* gene were strongly associated with ADFI and the highly correlated phenotype ADG. However, SNP located within the *NCAPG* gene were also associated with these traits making it difficult to definitively isolate which gene, if indeed only one, was implicated in these phenotypes. The data from the current study suggests that both genes may contribute depending upon the sex or stage of life of cattle.

While sex and maturity may play an important role in the expression of *NCAPG* and *LCORL*, it is possible that these differences are the result of nutritional or environmental effects. The diets fed to heifers, cows and steers vary in the amount of energy with steers receiving a high concentrate diet and the female populations on a high forage diet. The steers were the only group that did not show an association between *LCORL* in adipose tissue and ADFI. *LCORL* regulation in adipose may be the result of sex and/or tissue responses related to the type of feed, or it is possible that our steer population (n = 32) was not large enough to detect a significant effect. Whether in the muscle or adipose tissue, *LCORL* appears to be involved in pathway(s) that have an effect on or are affected by the amount of feed an animal consumes. As there is some evidence to suggest that the expression level of *NCAPG* may be more highly correlated to growth in a young, rapidly growing animals [5], and because we detected an association between gain and *NCAPG* in the muscle tissue of cows during active realimentation, it may be relevant to investigate the transcript abundance of these genes in the muscle tissue of young developing heifers in the future.

While the steer expression data from this study and the SNP associations from our previous study [4] suggest that *LCORL* is involved in feed intake and growth in the muscle tissue of steers, this trend does not appear to extend to the cow population. In fact, evidence in this study indicates that *NCAPG* rather than *LCORL* may have a role in the muscle tissue of cows experiencing a growth phase during an *ad libitum* feeding trial.

## Conclusions

The populations of animals examined in this study vary according to sex, age and diet, and it is plausible that any of these factors or a combination of these factors may be crucial for gene expression of either *NCAPG* or *LCORL* in adipose and muscle. Our data indicate that for steers in a feedlot setting, *LCORL* in muscle is contributing to or responding to the variation in feed intake. However, the expression of *LCORL* related to feed intake in female animals appears to occur in the adipose tissue. And contrary to steers, the *NCAPG* transcript abundance within the muscle of adult female cows is associated with the ADG phenotype. These differences suggest roles for both gene products in the muscle and adipose tissues of beef cattle in ADFI and ADG phenotypes, and may provide insight into the many SNP association and GWAS studies that have found association of one or both genes with growth, feed intake, height, length, carcass weight and carcass composition phenotypes.
